# Variable DNA methylation of aging-related genes is associated with male COPD

**DOI:** 10.1186/s12931-019-1215-7

**Published:** 2019-11-04

**Authors:** Xizi Du, Lin Yuan, Mengping Wu, Meichao Men, Ruoxi He, Leyuan Wang, Shuangyan Wu, Yang Xiang, Xiangping Qu, Huijun Liu, Xiaoqun Qin, Chengping Hu, Ling Qin, Chi Liu

**Affiliations:** 1 Department of Physiology; China-Africa Infection Diseases Research Center, Xiangya School of Medicine, Central South University, Changsha, 410078 Hunan China; 2 Department of Respiratory Medicine, National Clinical Research Center for Respiratory Diseases, Xiangya Hospital, Central South University, Changsha, Hunan China; 3 Health Management Center, Xiangya Hospital, Central South University, Changsha, Hunan China

**Keywords:** DNA methylation, Aging, COPD, Aging-related genes

## Abstract

**Background:**

Chronic obstructive pulmonary disease (COPD) is a chronic lung inflammatory disease which has a close relationship with aging. Genome-wide analysis reveals that DNA methylation markers vary obviously with age. DNA methylation variations in peripheral blood have the potential to be biomarkers for COPD. However, the specific DNA methylation of aging-related genes in the peripheral blood of COPD patients remains largely unknown.

**Methods:**

Firstly, 9 aging-related differentially expressed genes (DEGs) in COPD patients were screened out from the 25 aging-related genes profile through a comprehensive screening strategy. Secondly, qPCR and multiple targeted bisulfite enrichment sequencing (MethTarget) were used to detect the mRNA level and DNA methylation level of the 9 differentially expressed genes in the peripheral blood of 60 control subjects and 45 COPD patients. The candidate functional CpG sites were selected on the basis of the regulation ability of the target gene expression. Thirdly, the correlation was evaluated between the DNA methylation level of the key CpG sites and the clinical parameters of COPD patients, including forced expiratory volume in one second (FEV1), forced expiratory volume in one second as percentage of predicted volume (FEV1%), forced expiratory volume/ forced vital capacity (FEV/FVC), modified British medical research council (mMRC) score, acute exacerbation frequency and the situation of frequent of acute aggravation (CAT) score. Lastly, differentially methylated CpG sites unrelated to smoking were also determined in COPD patients.

**Results:**

Of the 9 differentially expressed aging-related genes, the mRNA expression of 8 genes were detected to be significantly down-regulated in COPD group, compared with control group. Meanwhile, the methylated level of all aging-related genes was changed in COPD group containing 219 COPD-related CpG sites in total. Notably, 27 CpG sites of FOXO3 gene showed a lower False Discovery Rate (FDR) and higher methylation difference values. Also, some variable DNA methylation is associated with the severity of COPD. Additionally, of the 219 COPD-related CpG sites, 147 CpG sites were not related to smoking.

**Conclusion:**

These results identified that the mRNA expression and DNA methylation level of aging-related genes were changed in male COPD patients, which provides a molecular link between aging and COPD. The identified CpG markers are associated with the severity of COPD and provide new insights into the prediction and identification of COPD.

## Background

Chronic obstructive pulmonary disease (COPD) is a major incurable chronic lung disease which is characterized by persistent, progressive airflow obstruction and increased inflammatory response in the airways [1]. It is predicted that COPD will become the third largest cause of death and bring global health burden in the world by 2020 in terms of the morbidity, mortality and economic burden [2]. Especially, the clinical treatment options for COPD are limited and largely ineffective, though a plethora of hypotheses have been proposed, which may be involved in the pathogenesis of COPD [3]. Several clinical researches support that accelerated lung aging is an important pathogenic mechanism of COPD [4, 5]. Epidemiological data also show that COPD preferentially affects elderly individuals, and people older than 65 have a higher incidence [6]. Notably, due to differences in risk factors exposure and smoking rate, COPD is more common in male patients in China [7, 8]. COPD, especially emphysema, has been shown to be associated with the acceleration of normal lung aging [9]. It is particularly noteworthy that all hallmarks of accelerated aging, such as telomere shortening, cellular senescence and stem cell exhaustion, were detected in COPD patients [10, 11]. It’s also evidenced that the level of anti-aging molecules such as histone deacetylases and sirtuins are reduced in COPD [11]. Anti-aging intervention can improve lung inflammation and COPD progression [12]. There has been a focus of interest on the mechanisms of aging and its role in the development of COPD. A series of recent evidences suggest that epigenetic mechanisms might be involved in the regulation of aging-related gene expression [13–15]. Different with traditional genetics, epigenetics involves alterations of genome information and interpretation, without alterations of nucleotide sequence. DNA methylation is the most intensively studied epigenetic mark in aging related studies [16, 17]. Differential methylation plays an important role in gene regulation and is further associated with the clinically phenotypic changes of different diseases [18]. More specifically, human aging is associated with DNA methylation changes at specific sites in the genome [19]. Age-related changes in DNA methylation has been detected in whole-blood or other different cell types, which revealed a large number of aging-related hyper- or hypo-methylation variation [20, 21]. However, there is no clear information on DNA methylation changes of aging-related genes in COPD patients. In this study, aging-related genes are defined as the genes that are changing persistently and consistently during aging process [22]. Firstly, we screened 25 aging-related genes through a certain screening strategy. Four COPD related datasets were used to prescreen out the differentially expressed aging-related genes in COPD. Then, we verified the mRNA expression of these aging-related genes and detected the DNA methylation level of the candidate CpG sites which may regulate the corresponding aging-related genes. Finally, we assessed the correlation between the DNA methylation level of the key CpG sites and the clinical parameters of COPD patients, including FEV1, FEV1%, FEV/FVC, mMRC score, acute exacerbation frequency and CAT score.

## Methods

### Aging datasets and COPD datasets selection

Aging datasets were searched in National Centre for Biotechnology Information’s Gene Expression Omnibus (GEO), using “aging”, “senescence” as keywords. The inclusion criteria were: (1) Genome or mRNA chip types; (2) Whole blood or PBMC sample; (3) Sample size > 30. The exclusion criteria were: (1) Lack of clear age description for samples; (2) Subjects have specific disease or received intervention during the studies. Three COPD datasets were identified in GEO using search terms including “COPD”, “whole blood” and “peripheral blood” (or combinations thereof). The inclusion criteria were: (1) Genome / mRNA chip types; (2) Study published in recent 3 years (2015–2017). The exclusion criteria were: (1) Lack of a specific definition of COPD; (2) Sample size is < 30; (3) Gene expression data is not available. GSE22148 was added to the COPD databases as it has the same cohort source as GSE76705 and the importance of induced sputum in the pathogenesis of COPD. The summary of these datasets was shown in Table 1.

**Table 1:** Summary of aging-related datasets and COPD-related datasets

Data set	Sample type	Group	Gender
Aging-related dataset GSE58137	Whole blood	15–78 years of age (359)	Female/Male
Aging-related dataset GSE65219	PBMC	19–90 years of age (176)	Female/Male
Aging-related datasets GSE47728	Whole blood	44–87 years of age (228)	Female/Male
COPD-related dataset GSE56768	Whole blood	COPD (49) / Control (31)	Female/Male
COPD-related dataset GSE42057	PBMC	COPD (94) / Control (42)	Female/Male
COPD-related dataset GSE76705	Whole blood	COPD (141) / Control (88)	Female/Male
COPD-related dataset GSE22148	Inflammatory cells from induced sputum	Severe COPD (103) / Moderate COPD (71)	Female/Male

### Differentially expressed aging-related genes analysis in COPD datasets

Differential gene expression with increasing age was analyzed using an adjusted linear regression model in aging datasets (*p*-value < 0.05). The overlapped genes between three datasets would be screened out. Based on whether it is expressed in lung tissue and related literature data, candidate aging-related genes were further identified. Then, to integrate data from 4 microarray and identify DEGs in COPD, a meta-analysis was conducted using Revman 5.3 software. Effect size and corresponding standard error were obtained from each individual study. Besides, *p*-value < 0.05 was selected as threshold and study heterogeneity was tested by using the X^2^ test and I^2^ statistics.

### Cohorts and phenotypes

The protocols were approved by No. 201705820 of the Xiangya Hospital Ethics Review Committee and all subjects were provided with written consent. Study subjects were selected from the Respiratory Department and the Medical Examination Center of Xiangya Hospital, Changsha. The samples and data were collected from June 2017 to August 2018, including questionnaire information (general condition, smoking history, clinical symptoms of COPD, other respiratory diseases, acute exacerbations in past 12 months, CAT score), pulmonary function testing and peripheral blood samples. The inclusion criteria for the case group were men between the age of 40 and 70 with a clear diagnosis of COPD (meeting the standard of GOLD: 2017 global strategy for the diagnosis, management and prevention of COPD) and no other diseases (e.g. other respiratory and cardiovascular diseases, diabetes). For our analysis, lung function phenotypes were used included the spirometric values of FEV1 and the ratio of FEV1 to the FVC. COPD was defined as present when the FEV1/FVC ratio < 0.7 and FEV1% < 70%. The control group was in the same age and gender without COPD definition or other acute or chronic diseases, including smoking controls and non-smoking controls. Strict quality control measures were implemented. All interviews and examinations were performed by certified staff. Moreover, regular feedback about the quality of their performance was given to each field worker during data collection, and retraining was undertaken when necessary.

### Quantitative real-time PCR

The total RNA of the peripheral blood samples was extracted by Trizol (Invitrogen) and quantified on a SmartSpec™ Plus spectrophotometer (Bio-RAD, USA). cDNA synthesis was performed with 1 μg of total RNA in a 20 ul reaction mix system by use of PrimeScript™ RT Master Mix Kit (Takara, Japan). Quantitative real-time PCR (qPCR) was performed on a CFX96 Touch™ Deep Well Real-Time PCR Detection System (Bio-RAD, USA) by use of TaqMan Gene Expression Master Mix (Applied Biosystems) with thermal cycling conditions. Primer sequences of target gene were described in Additional file 1. Resulting mRNA levels were normalized to β-actin and expressed as a fold change relative to control samples.

### DNA extraction, bisulfite treatment, methylation Array methods

Due to the simplicity of the clinical procedures involved, epigenetic changes identified in blood can represent other tissues that are valuable for diagnostic and prognostic biomarkers. We collected peripheral blood from 45 COPD cases and 60 controls for Multiple targeted bisulfite enrichment sequencing (MethTarget). DNA extraction and quality control, bisulfite processing, methylation library construction and high-throughput sequencing were carried out at Genesky Biotechnologies Inc. Shanghai [23]. Briefly, Genomic DNA was extracted from whole blood with commercially available kits (TIANGEN Biotech, Beijing, China) according to previous publications [24, 25]. Purified DNA was quantified and then diluted to a working concentration of 10 ng/ul for genotyping. CpG islands located in the proximal promoter of targeted genes were selected for measurement according to the following criteria: (1) 200 bp minimum length; (2) 50% or higher GC content; (3) 0.60 or higher ratio of observed / expected dinucleotides CpG. Finally, 47 regions from CpG islands of targeted gene were selected and sequenced (7 from AREG, 4 from ATG3, 4 from E2F1, 12 from FOXO3, 2 from HDAC1, 4 from MMP2, 4 from NUF2, 6 from TGFB1, 4 from TP53). On this basis, bisulfite modification of DNA sample was performed with EZ DNA Methylation-Gold Kit (ZYMO, CA, USA). Library was constructed subsequent to multiplex PCR reaction. The product was sequenced in the Illumina MiSeq Benchtop Sequencer (CA, USA). After bisulfite treatment, the primer sequences used for qPCR were designed by primer 3 (http://primer3.ut.ee/). A total of 939 CpG sites from the 9 differentially expressed aging-related genes were detected in the methylation assay. We only retained the raw data with a sequencing quality value Q > 40 (Base sequencing error rate < 0.1%) and reported the percent methylation of every CpG site.

### Statistical analysis

The characteristic data of recruited COPD patients and controls were presented as Mean ± SD, *p*-value < 0.05, analyzed by unpaired T test. The mRNA expression and the methylation array of aging-related genes were analyzed by T test and nonparametric test (Mann-Whitney U test). The method of Benjamin Hochberg was used to control the false discovery rate (FDR). Logistic regression analysis was performed on selected differentially expressed CpG sites, with potential risk factor of age, smoking history, work environment and outdoor pollution [26]. Pearson’s correlation was used to assess the association between the percentage of methylation of differentially expressed CpG sites and the continuous variables such as FEV1, FEV1%, FEV1/FVC. Ordinal categorical variables, such as mMRC score, were accessed by Spearman’s correlations which were also used for association analysis between age and age-related genes. The predictive accuracy of differentially expressed CpG sites to the severity of COPD was compared with CAT score and the definitions of acute aggravation. The area under the receiver operating characteristic (AUC/ROC) curves was used to evaluate the accuracy. Statistical analysis was done in R language and SPSS.19 software. A two-tailed *p*-value < 0.05 was considered statistically significant.

## Results

### Screening of differentially expression aging-related genes in COPD patients

The specific strategies for the selection of the 9 differentially expression aging-related genes were shown in a flow chart (Additional file 2). First, 128 aging-related genes were selected from three aging-related datasets through comprehensive protocol. Only 78 genes that are highly expressed in lung are further screened out by Gene (gene-centered information at NCBI) from the 128 genes. Then, 25 differential expression genes were selected as the aging-related genes profile, which, based on previous literatures, were less studied in COPD or considered to be classical ageing-related gene [27–32]. In addition, based on the 4 selected COPD-related databases, meta-analysis of the candidate 25 aging-related genes showed that 9 genes (FOXO3, TP53, TGFβ1, MMP2, HDAC1, NUF2, ATG3, AREG and E2F1) were significantly altered in the COPD group compared to the control group (Table 2 and Additional file 3). Furthermore, we also performed an association analysis of age and age-related genes mRNA level in non-smoking controls. The result showed that the expression of these screened genes decreased with age (Spearman’ s correlations, *p*-value < 0.05) which was showed in Additional file 4.

**Table 2:** Meta-analysis of 9 different expressed aging related genes in COPD group

Gene symbol	Effect size (SMD)	95% CI	*p*-value
TGFB1	− 3.85	[−6.38, − 1.31]	0.003
TP53	−2.66	[−4.44, − 0.88]	0.003
MMP2	−1.76	[−2.96, −0.56]	0.004
AREG	−3.02	[−5.25, −0.80]	0.008
E2F1	−1.4	[−2.51, −0.30]	0.01
HDAC1	−3.3	[−5.94, −0.67]	0.01
NUF2	−2.62	[−4.72, −0.52]	0.01
FOXO3	−1.84	[−3.34, −0.34]	0.02
ATG3	−2.6	[−4.89, −0.30]	0.03

A *p* value < 0.05 was considered statistically significant

### Decreased expression of aging-related genes in COPD patients

**Fig. 1:**
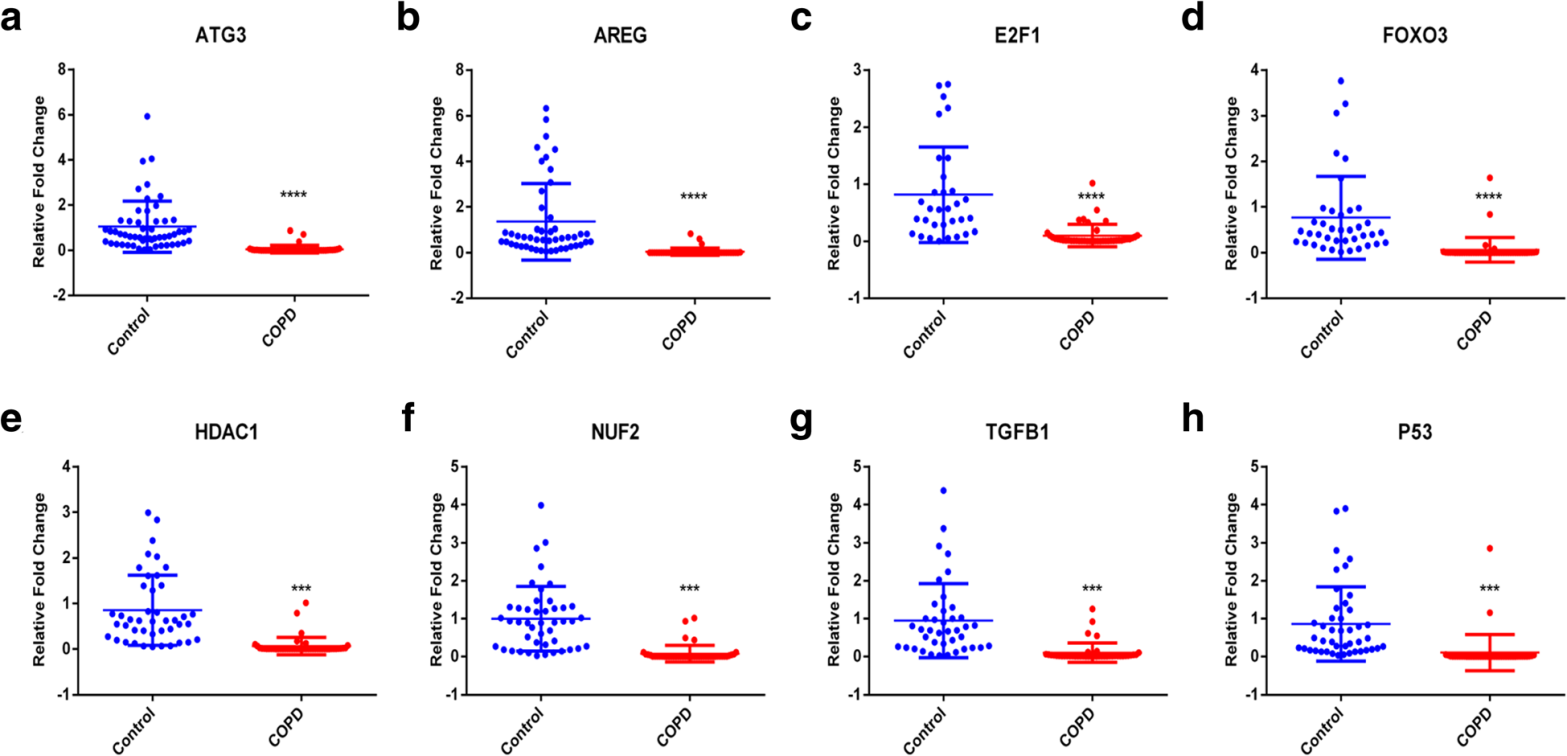
The mRNA level of the aging-associated genes FOXO3, TP53, TGFβ1, MMP2, HDAC1, NUF2, ATG3, and AREG were significantly down-regulated in peripheral blood of COPD patients. (**a**-**h**) The mRNA expression of these genes was detected by qPCR. *** *p* < 0.001; **** *p* < 0.0001

In order to further verify the differential expression of the selected genes in public datasets, we recruited 45 COPD patients and 60 controls. All selected subjects are male. The demographic characteristics of recruited subjects are presented in Table 3. There was no statistic difference in age between groups. Among all the 9 genes, the mRNA expression of FOXO3, TP53, TGFβ1, HDAC1, NUF2, ATG3, AREG and E2F1 was significantly down-regulated in the COPD group compared with the control group (Fig. 1), which was consistent with previous meta-analysis results. While, the mRNA expression of MMP2 was too low to be detectable both in the COPD group and the control group.

**Table 3:** Demographic Characteristics of the COPD patients and controls

			Control	COPD
Number of subjects			60	45
Age			55.26 ± 7.14	60.58 ± 6.61
Smoking history (Yes/No)			25/35	45
FEV1			2.622 ± 0.1438	1.149 ± 0.1017*
FEV1%predicted			92.47 ± 9.26	39.52 ± 18.11*
FEV1/FVC			84.08 ± 8.85	41.1 ± 13.36*
Acute exacerbation frequency (Y/N)			/	15/30
mMRC score		0/1/2/3/4	/	2/8/12/15/9
CAT score		< 10/> 10	/	12/34

Data are presented as Mean ± SD, *p < 0.05, COPD patients VS controls (Unpaired t test). FEV1 - forced expiratory volume in 1 s, presented as absolute volume and percentage of predicted volume (FEV1%); FVC - forced vital capacity; mMRC – modified British medical research council; CAT – COPD assessment test; the situation of acute aggravation frequency was judged by acute attack more than twice in last 12 months

### Methylation change of aging-related genes in COPD patients

**Fig. 2:**
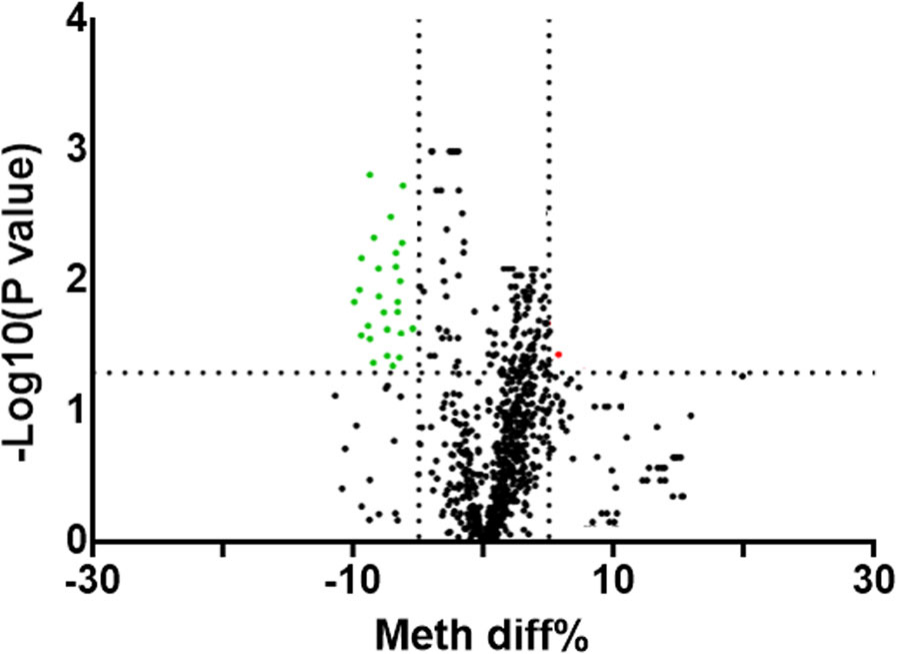
Volcano plot of differential methylation CpG sites between COPD patients and normal volunteers. The up-regulated sites were presented as red dots and down-regulated as green. *p*-value< 0.05

Since some aging-related genes have shown to be down-regulated in COPD patients, we further want to determine whether these down-regulated aging-related genes could be mediated by DNA methylation. DNA was extracted to detect 939 CpG sites in all the 9 aging-related genes. The differential methylation analysis was represented using volcano maps (Fig. 2). 27 CpG sites were associated with COPD, and all of them appeared in the sequence of FOXO3 gene at FDR < 5% and absolute value of meth diff > 5%. Then, we further set the standard at FDR < 5%, and the absolute value of meth diff > 0.1%. Under such screening conditions, 219 COPD-related CpG sites appeared in all 9 tested genes. The absolute value of methylation difference between the top five sites ranged from 0.33 to 9.09%. To further investigate the effect of differentially expressed CpG sites’ on disease status, a logistic regression analysis with potential risk factors adjustment was performed. Among 219 sites, 191 sites remained statistic significant after logistic regression analysis, while other 28 sites not. This was under a condition that the methylation expression is analyzed as a continuous variable. However, all methylation sites were significant different when we grouped methylation expressions according to quartiles and analyzed as four categorical variables. The top 2 differentially methylated CpG sites of each tested gene were also shown in Table 4. Besides, the complete list of the differentially methylated CpG sites is provided in Additional file 5.

**Table 4:** The top 2 differentially methylated CpG sites associated with COPD

CpG Site	Gene	Mean Difference Methylation	FDR Adjusted *p*-value	Logistic regression model for risk factors	
				Adjust β (95% CI)	*p*-value
chr4:75310999	AREG	0.46%	0.011	71.34(1.79, 140.9)	0.044
chr4:75310908	AREG	0.45%	0.008	49.92(−3.04, 102.88)	0.065
chr3:112281685	ATG3	−2.61%	0.037	−8.45(−16.32, −0.59)	0.035
chr3:112281810	ATG3	−1.83%	0.037	− 12.00(−23.35, − 0.64)	0.038
chr6:108882977	FOXO3	−9.09%	1.68855E-05	−8.83(− 14.65, −3.00)	0.003
chr6:108882982	FOXO3	−9.04%	3.652E-05	−8.52(−14.26, −2.78)	0.004
chr20:32273763	E2F1	−3.45%	0.008	−21.5(−43.20,0.19)	0.052
chr20:32274387	E2F1	2.72%	0.014	20.01(4.35, 35.66)	0.012
chr1:32757717	HDAC1	0.67%	0.026	29.61(0.34, 58.89)	0.047
chr1:32757818	HDAC1	−0.41%	0.027	−50.71(−97.74, −3.65)	0.035
chr1:163292017	NUF2	−0.42%	0.011	−97.07(− 171.02, −23.11)	0.10
chr1:163291916	NUF2	0.33%	0.005	67.50(−5.96, 140.98)	0.072
chr16:55514378	MMP2	−1.66%	0.013	−14.01(−27.68, −0.34)	0.045
chr16:55514466	MMP2	−1.50%	0.023	−16.59(− 33.14, −0.05)	0.049
chr19:41859656	TGFB1	1.82%	0.033	11.88(1.38, 22.37)	0.027
chr19:41859677	TGFB1	−1.67%	0.01	−34.21(−60.97, − 7.45)	0.012
chr17: 7591645	TP53	−3.83%	0.001	139.19(18.45, 257.93)	0.024
chr17:7590743	TP53	−0.68%	0.026	209.32(80.13, 338.51)	0.001

Differential methylation analysis was conducted between COPD patients and controls in blood samples from a total of 105 subjects. The method of Benjamin Hochberg was used to control the false discovery rate (FDR), *p* < 0.05; Adjusted β were derived from Binary logistic regression analysis. These factors were adjusted in the logistic regression analysis: age, smoking history and work environment and outdoor pollution

### Potential correlation between aging-related genes and COPD variables

**Fig. 3:**
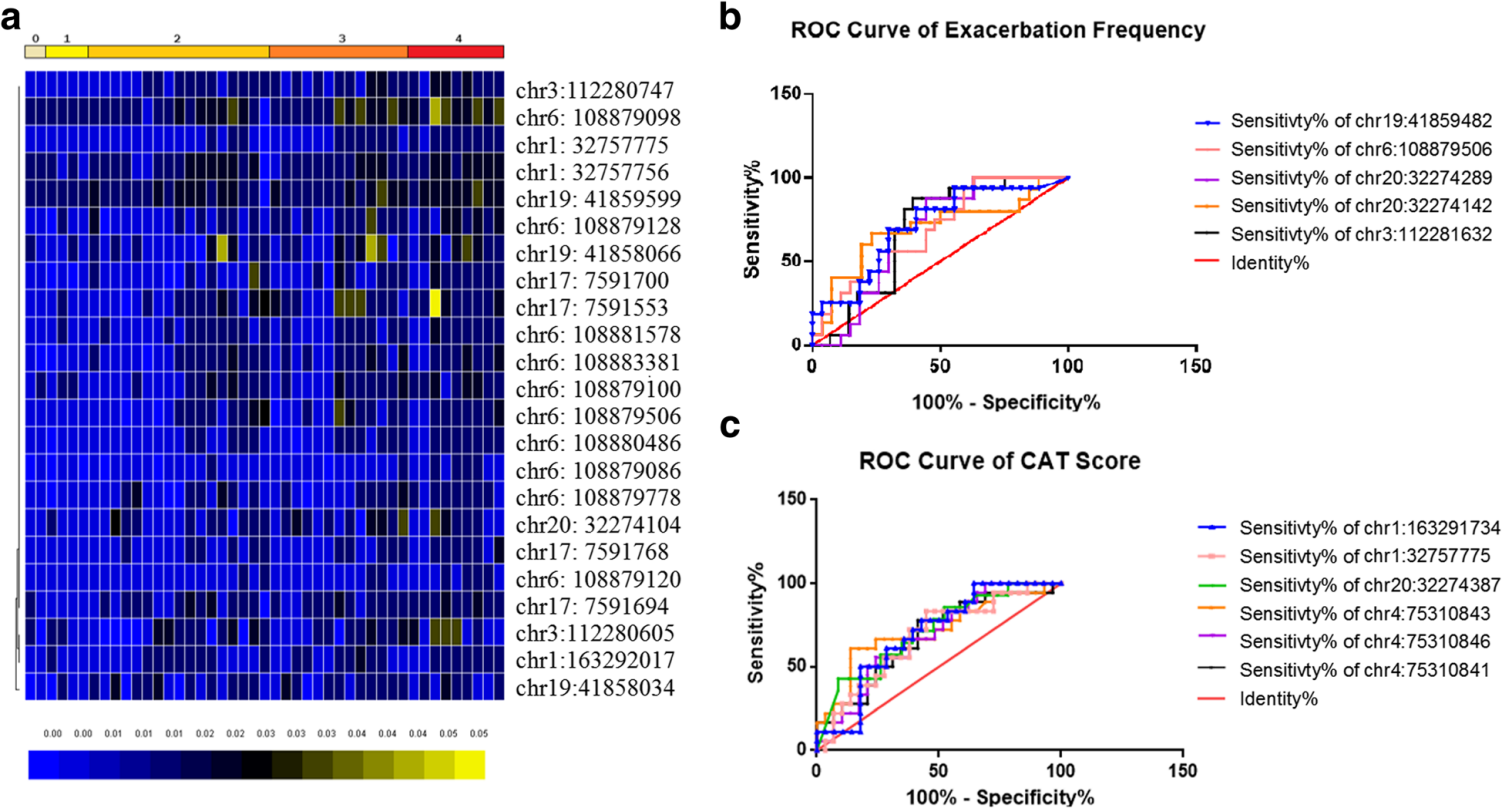
Correlation analysis between differential methylation sites and other clinical indicators. Correlation analysis between differential methylation sites and clinical indicators. **a**. CpG sites positively associated with the mMRC score (0–4). *p*-value< 0.05. The methylation differences at 23 sites between different mMRC groups are presented in the clustering map. **b** ROC curve of acute exacerbation frequency *p*-value < 0.05, AUR > 0.5. **c** ROC curve of CAT score. *p*-value < 0.05, AUR > 0.5

To further understand whether the epigenetic changes of aging-related genes are correlated to the progression and severity of COPD, we analyzed the correlation between the differentially methylated CpG sites and the clinical indicators of COPD patients. Firstly, no significant correlation was found between the lung function indicators FEV1%, FEV1, FEV/FVC and the differentially methylated CpG sites. Next, mMRC is classified into 0–4 grades based on the patient’s activity during shortness of breath, with grade 4 indicates the patient shows the slightest activity. Twenty three differentially methylated CpG sites were positively correlated with the mMRC score by rank correlation analysis. The methylation differences at 23 sites between different mMRC groups are presented in the clustering map (Fig. 3a). The predictive accuracy of differentially expressed CpG sites to COPD severity was assessed by CAT score comparison (> 10/< 10) and acute exacerbation frequency (acute attack more than twice per year or not). The ROC curve of acute exacerbation frequency showed that there were 5 sites with potential predictive significance, including chr19:41859482 (TGFB1), chr6:108879506 (FOXO3), chr20:32274289 (E2F1), chr20:32274142 (E2F1) and chr3:112281632 (ATG3). The areas under the curve are respectively 68.8, 69.5, 68.3, 69.4 and 71.2% (Fig. 3b). The ROC curve of CAT score shows that there are still 6 CpG sites with potential diagnostic significance (chr1:163291734, chr1:32757775, chr20:32274387, chr4:75310843, chr4:75310846, chr4:75310841), which belong to NUF2, HDAC1, E2F1 and AREG gene. The corresponding areas under the curve are 68.1, 71.6, 68.2, 71.6, 67.8 and 69.8%, respectively (Fig. 3c). Two CpG sites, chr6:108879506 (FOXO3) and chr1:32757775 (HDAC1) were obtained when we compared the meaningful CpG sites derived from the mMRC score to that derived from the CAT score or the acute exacerbation.

### Effect of external factors other than smoking on methylation changes in aging-related CpG sites

**Fig. 4:**
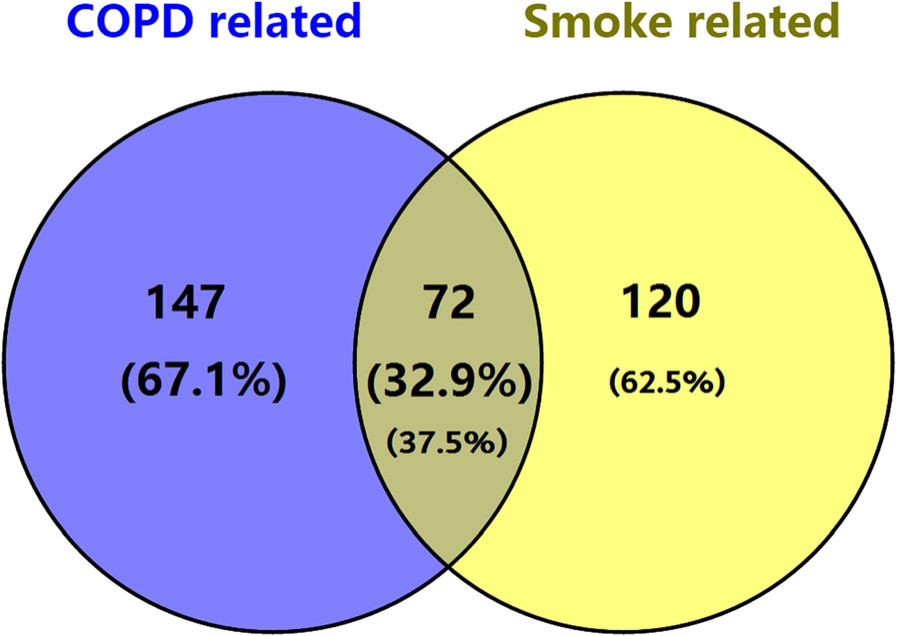
Venn diagram of the intersection of aging-related CpG sites and smoking-related CpG sites

Smoking is the major environmental contributor to COPD. Cigarette smoke-induced DNA methylation may also relate to the initiation and progression of COPD. However, the altered methylation sites of aging-related genes may be not solely caused by smoking. To assess the possible impact of other factors (except smoking) in the methylation regulation of aging-related genes, we compared the altered CpG sites between healthy smoker group (25) and non-smoker group (35). Of the 219 COPD related methylation sites, only 72 coincided with smoking-related methylation sites. There are still 147 COPD-related CpG sites that are not associated with smoking (Fig. 4, Additional file 5). This differential methylation CpG site distribution indicates that the methylation changes of these aging-related genes in COPD group are only partly caused by smoking.

## Discussion

COPD is a common chronic lung disease which has an irreversible process that contributes increasingly to the global health burden [33]. Many studies have shown that aging acceleration of lung is engaged in the pathogenesis of COPD [34, 35]. In recent few years, the epigenetic alterations have gained increasing attention as an important influencing factor of aging [36]. DNA methylation has also been verified to be the most in-depth epigenetic marker in aging study which is specific to cytosine, especially to CpG dinucleotides [37]. The CpG site associated with aging is either hypermethylated or hypomethylated in the process of aging [27, 38]. In this study, we further probe the methylation alteration of aging-related genes in COPD patients. At first, we screened 25 aging-related genes that were not fully studied or considered to be classical aging-related genes through a certain screening strategy. Then, differentially expressed aging-related genes in COPD patients were screened out through COPD databases. Specifically, based on the 4 selected COPD databases, 9 differentially expressed aging-related genes in COPD patients were identified with meta-analysis from the aging-related gene profile, including AREG, ATG3, E2F1, FOXO3, NUF2, HDAC1, MMP2, TP53 and TGFβ1. The role of these 9 genes in the regulation of aging through different regulatory mechanisms has been studied to varying degrees [28, 39–46]. And some of these aging-related genes have been verified to be a key regulator of COPD [40, 47–49]. Other than those genes that have been clearly involved in the pathogenesis of COPD, the role of AREG, ATG3, HDAC1, NUF2 and E2F1 in the pathogenesis of COPD has rarely been investigated. Notably, the mRNA expression of almost all genes was significantly down-regulated in the peripheral blood of COPD patients except for MMP2. One possible reason is that the expression of MMP2 is too low to be detectable both in healthy controls and COPD patients. Decreased expression of ATG3, FOXO3, HDAC1 and NUF2 in COPD patients was consistent with previous studies [40, 50–52]. Although there is still debate about the expression of E2F1 and TGFβ1 which were down-regulated in the peripheral blood of COPD patients, the results of AREG and TP53 were opposite to the previous results. The expression of AREG was up-regulated in primary bronchial epithelial cells, which was different from the decreased expression in peripheral blood. Surprisingly, the classical aging-related protein TP53 was also down-regulated in COPD patients. It has been observed that TP53 deletion aggravated elastase-induced emphysema severity, pulmonary inflammation and lung oxidative stress [53]. Then, we speculated that the down-regulation of TP53 expression in peripheral blood may be related to the severity of the recruited COPD patients. Further, the methylation status of CpG sites from the 9 gene had been detected. Although MMP2 mRNA expression were not detected from whole blood sample, it is not appropriate to completely deny its effect on COPD. Consistent with the mRNA expression, the methylation level of the most sites was up-regulated in COPD patients. These results suggested that the expression changes of these aging-related genes were partly related to the regulation of DNA methylation. Moreover, correlation analysis also showed that 23 differentially methylated CpG sites were positively correlated with the mMRC score in assessing the degree of dyspnea. Two coincident sites (chr6:108879506 and chr1:3275775) were obtained when we compared the meaningful CpG sites derived from the mMRC score to that derived from the CAT score or the acute exacerbation. However, there was no correlation between lung functional assessment indicators (FEV1%, FEV1 and FEV/FVC) and methylation level of differential CpG sites. Although DNA methylation of aging-related gene may be a cause of COPD exacerbation, it may not certainly correspond to a specific clinical change due to the hysteresis effect of epigenetic modifications. Apart from this, there are many other factors can influence the progression of COPD. Since we currently do not have long enough follow-up to examine the methylation alteration and changes of lung function, it is hard to determine whether methylation pattern of these aging-related genes is a cause or consequence in the pathogenesis of COPD.

In addition, 147 COPD-related CpG sites are not associated with smoking in all the 219 COPD related methylation sites, which indicates that the methylation changes of these aging-related genes in COPD group are only partly caused by smoking. However, there are more potential differences between these groups than merely smoking, which may interfere with our conclusions to some extent. It is also intriguing that we didn’t find CpG sites that coincided with previous EWAS of COPD. It may be partly due to different laboratory design, analytical methods and quality control (such as age, gender and ethnic differences) [54–56]. Although these specific CpG sites may provide potential diagnostic significance for the assessment of COPD severity, there are some limitations in this study. The first one is the lack of female COPD patients which may be different from the age-related DNA methylation patterns in man [57]. Besides, the regulation mechanism of these aging-related genes by DNA methylation and the possible application in practice still needs further exploration. Moreover, the selection strategy for aging-related genes is also not comprehensive enough which should be improved in subsequent studies.

## Conclusions

In summary, this study verified the differential expression of aging-related genes in peripheral blood of COPD patients, which may be regulated by DNA methylation. The methylation level of some specific CpG sites was associated with the prevalence and severity of COPD. These results provide some useful insights into the molecular mechanisms of aging in COPD and may also provide some valuable biomarkers for early diagnosis and prognosis of COPD.

## Supplementary information

**Additional file 1.** Primer sequence of aging-related genes for qPCR

**Additional file 2.** Flow chart of aging-related genes selection process

**Additional file 3.**Meta-analysis of 25 candidate aging-related genes in COPD group. *p*-value < 0.05 was considered as significant

**Additional file 4. **The expression of differentially expressed aging-related genes decreased with age. Non-smoking control samples showed a continues mRNA decrease in relation to age. Spearman’s correlation between mRNA level of aging-related genes (AREG, ATG3, E2F1, FOXO3, HDAC1, NUF2, TGFβ1 and TP53) and age is significant. Data are represented as scatter plots with linear fits. * *p* < 0.05; ** *p* < 0.01

**Additional file 5.** Differentially methylated CpG sites associated with COPD

## Data Availability

All data used and analyzed in this study are included in this article or are available in the GEO database (https://www.ncbi.nlm.nih.gov/geo/query/acc.cgi?acc=GSE16972; https://www.ncbi.nlm.nih.gov/geo/query/acc.cgi?acc=GSE13896; https://www.ncbi.nlm.nih.gov/geo/query/acc.cgi?acc=GSE22148; https://www.ncbi.nlm.nih.gov/geo/query/acc.cgi?acc=GSE56768; https://www.ncbi.nlm.nih.gov/geo/query/acc.cgi?acc=GSE42057).

## Abbreviations

CAT: the situation of frequent of acute aggravation
COPD: Chronic obstructive pulmonary disease
DEGs: Differentially expressed gene
FEV1: Forced expiratory volume in one second
FEV1%: Forced expiratory volume in one second as percentage of predicted volume
FVC: Forced vital capacity
GEO: Gene Expression Omnibus
mMRC: modified British medical research council
