# Supplementary material for: Variable DNA methylation of aging-related genes is associated with male COPD
Source: Respir Res. 2019 Nov 4;20:243. doi: 10.1186/s12931-019-1215-7 (PMC6829949; doi:10.1186/s12931-019-1215-7)
Supplement: Supplementary file 1 — Additional file 1. Primer sequence of aging-related genes for qPCR [file 12931_2019_1215_MOESM1_ESM.docx]

Additional file 1. Primer sequence of aging-related genes for qPCR.

| Gene | Primer |  |  |
| --- | --- | --- | --- |
| AREG | forward |  | TGTCGCTCTTGATACTCGGC |
|  | reverse |  | AGGCATTTCACTCACAGGGG |
| ATG3 | forward |  | GTGTTCAGTTCACCCATGCAG |
|  | reverse |  | TTAACAGCCATTTTGCCACTAATCT |
| HDAC1 | forward |  | CGATGGCCTGTTTGAGTTCTG |
|  | reverse |  | CCCTCTGGTGATACTTTAGCAGT |
| NUF2 | forward |  | TGTTAAGCAATACAAACGCACAG |
|  | reverse |  | TGCCTTTTCAATACCGTCGTG |
| E2F1 | forward |  | CATCCCAGGAGGTCACTTCTG |
|  | reverse |  | GACAACAGCGGTTCTTGCTC |
| FOXO3 | forward |  | CGGACAAACGGCTCACTCT |
|  | reverse |  | GGACCCGCATGAATCGACTAT |
| TGFB1 | forward |  | CGACTCGCCAGAGTGGTTAT |
|  | reverse |  | GCTAAGGCGAAAGCCCTCAA |
| MMP2 | forward |  | GATGGCAAGTACGGCTTCTG |
|  | reverse |  | GGTGCAGCTGTCATAGGATGT |
| TP53 | forward |  | AAGTCTGTGACTTGCACGTACTCC |
|  | reverse |  | GTCATGTGCTGTGACTGCTTGTAG |
