# Supplementary figures and images for: Variable DNA methylation of aging-related genes is associated with male COPD
Source: Respir Res. 2019 Nov 4;20:243. doi: 10.1186/s12931-019-1215-7 (PMC6829949; doi:10.1186/s12931-019-1215-7)

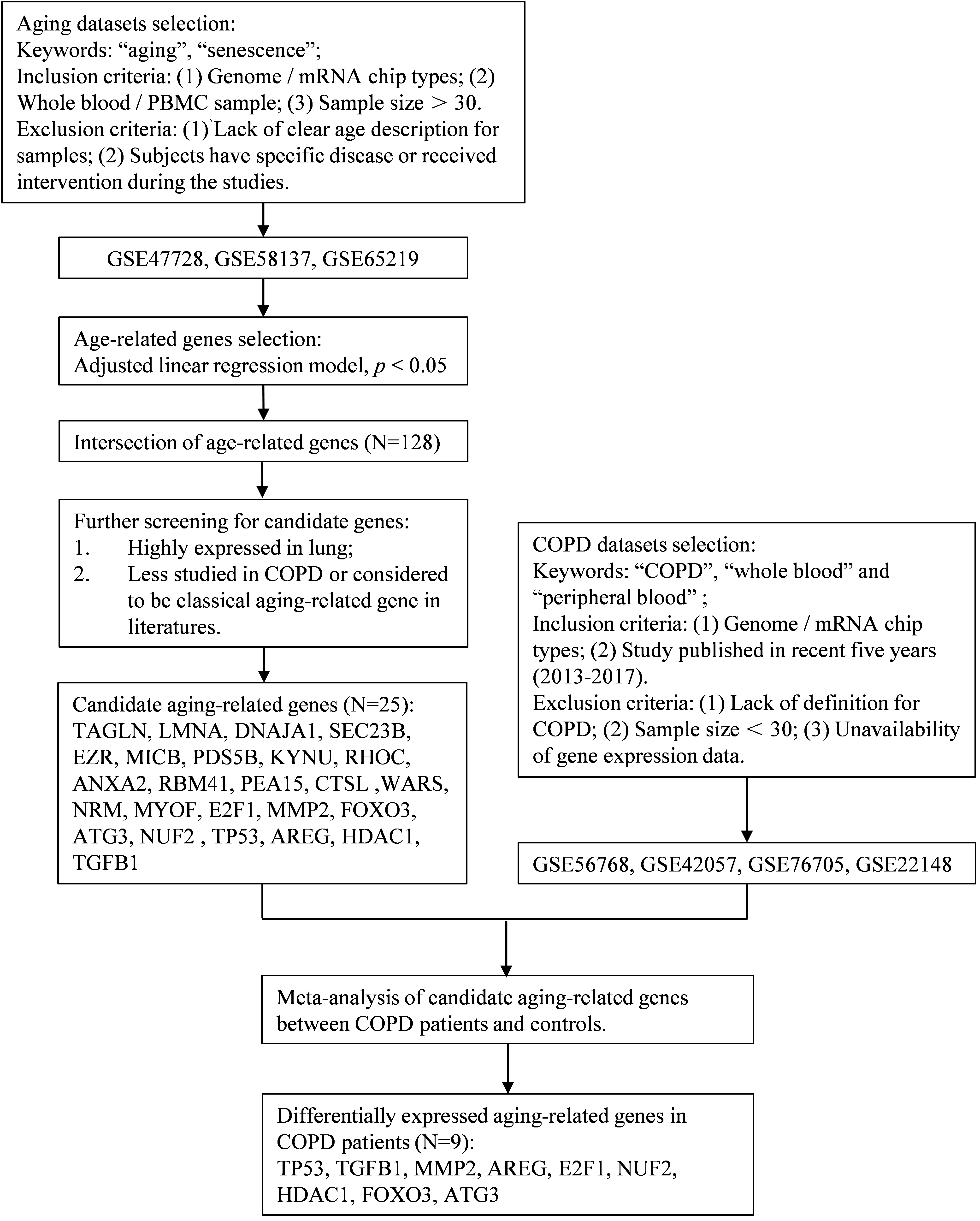

Supplement: Supplementary file 2 — Additional file 2. Flow chart of aging-related genes selection process [file 12931_2019_1215_MOESM2_ESM.tif]

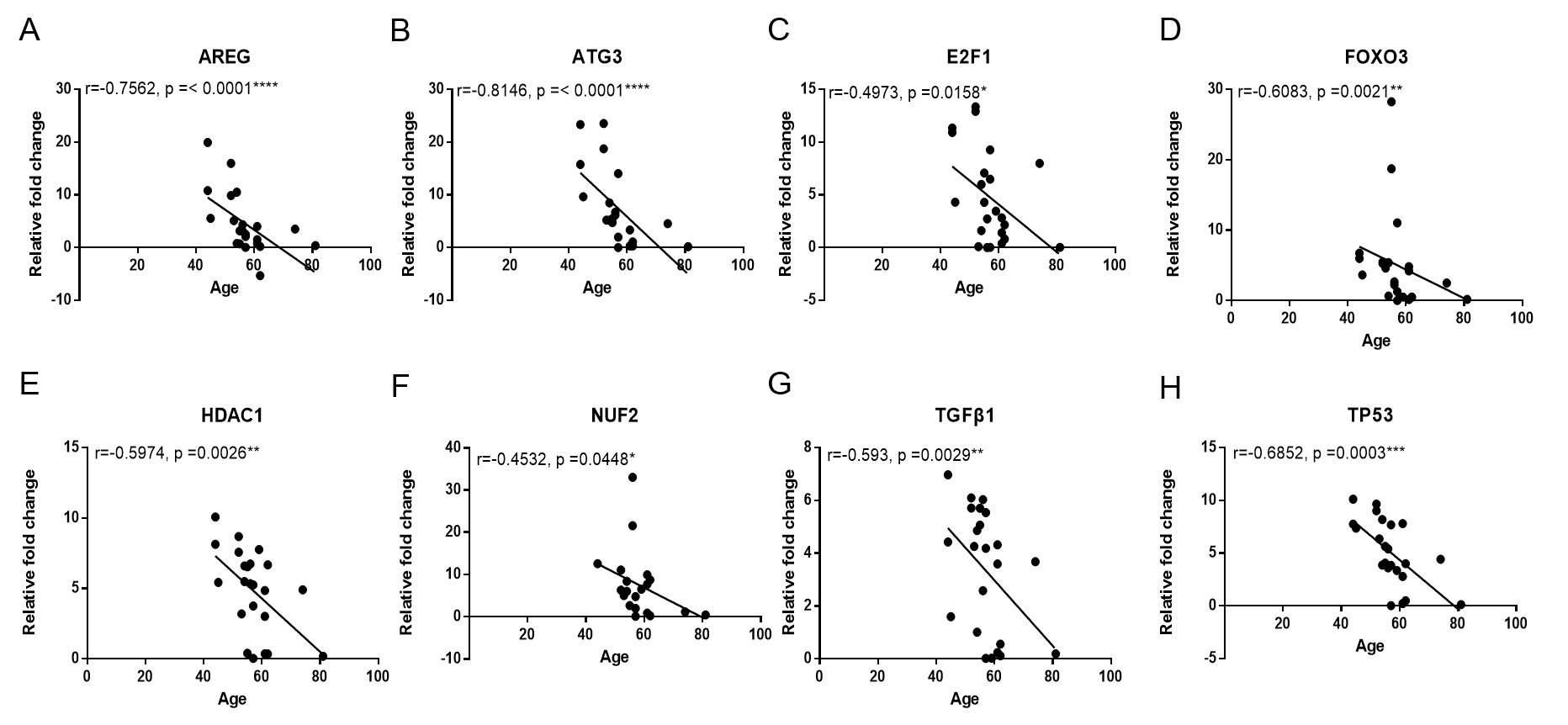

Supplement: Supplementary file 4 — Additional file 4. The expression of differentially expressed aging-related genes decreased with age. Non-smoking control samples showed a continues mRNA decrease in relation to age. Spearman’s correlation between mRNA level of aging-related genes (AREG, ATG3, E2F1, FOXO3, HDAC1, NUF2, TGFβ1 and TP53) and age is significant. Data are represented as scatter plots with linear fits. * p < 0.05; ** p < 0.01 [file 12931_2019_1215_MOESM4_ESM.tif]
