# Supplementary material for: Variable DNA methylation of aging-related genes is associated with male COPD
Source: Respir Res. 2019 Nov 4;20:243. doi: 10.1186/s12931-019-1215-7 (PMC6829949; doi:10.1186/s12931-019-1215-7)
Supplement: Supplementary file 3 — Additional file 3.Meta-analysis of 25 candidate aging-related genes in COPD group. p-value < 0.05 was considered as significant [file 12931_2019_1215_MOESM3_ESM.docx]

Additional file 3. Meta-analysis of 25 candidate aging related genes in COPD group.

| Gene symbol | Effect size (SMD) | 95%CI | *p*-value |
| --- | --- | --- | --- |
| \| TGFB1 \| \| --- \| \| TP53 \| \| MMP2 \| \| AREG \| \| E2F1 \| \| HDAC1 \| \| NUF2 \| \| FOXO3 \| \| ATG3 \| \| RHOC \| \| LMNA \| \| KYNU \| \| NRM \| \| DNAJA1 \| \| MYOF \| \| TAGLN \| \| SEC23B \| \| PEA15 \| \| PDS5B \| \| RBM41 \| \| EZR \| \| MICB \| \| CTSL \| \| WARS \| \| ANXA2 \| | \| -3.85 \| \| --- \| \| -2.66 \| \| -1.76 \| \| -3.02 \| \| -1.4 \| \| -3.3 \| \| -2.62 \| \| -1.84 \| \| -2.6 \| \| 0.51 \| \| 1.51 \| \| 0.64 \| \| -0.98 \| \| 1.09 \| \| -1.16 \| \| 1.57 \| \| 1.06 \| \| -0.34 \| \| 0.75 \| \| 0.13 \| \| 0.87 \| \| 0.77 \| \| -0.48 \| \| -0.51 \| \| 0.33 \| | \| [-6.38, -1.31] \| \| --- \| \| [-4.44, - 0.88] \| \| [-2.96, -0.56] \| \| [-5.25, -0.80] \| \| [-2.51, -0.30] \| \| [-5.94, -0.67] \| \| [-4.72, -0.52] \| \| [-3.34, -0.34] \| \| [-4.89, -0.30] \| \| [-0.07, 1.10] \| \| [-0.28, 3.30] \| \| [-0.27, 1.54] \| \| [-1.94, -0.02] \| \| [-0.63, 2.80] \| \| [-3.38, 1.06] \| \| [-2.76, 5.91] \| \| [-2.01, 4.13] \| \| [-1.39, 0.72] \| \| [-2.28, 3.78] \| \| [-0.43, 0.70] \| \| [-3.61, 5.36] \| \| [0.01, 1.53] \| \| [-3.07, 2.12] \| \| [-3.45, 2.43] \| \| [-1.76, 2.41] \| | \| 0.003 \| \| --- \| \| 0.003 \| \| 0.004 \| \| 0.008 \| \| 0.01 \| \| 0.01 \| \| 0.01 \| \| 0.02 \| \| 0.03 \| \| 0.08 \| \| 0.1 \| \| 0.17 \| \| 0.18 \| \| 0.22 \| \| 0.31 \| \| 0.48 \| \| 0.5 \| \| 0.53 \| \| 0.63 \| \| 0.65 \| \| 0.7 \| \| 0.7 \| \| 0.72 \| \| 0.73 \| \| 0.76 \| |
|  |  |  |  |

A *p* value < 0.05 was considered statistically significant.
