# Supplementary material for: Variable DNA methylation of aging-related genes is associated with male COPD
Source: Respir Res. 2019 Nov 4;20:243. doi: 10.1186/s12931-019-1215-7 (PMC6829949; doi:10.1186/s12931-019-1215-7)
Supplement: Supplementary file 5 — Additional file 5. Differentially methylated CpG sites associated with COPD [file 12931_2019_1215_MOESM5_ESM.docx]

Additional file 5. Differentially methylated CpG sites associated with COPD.

| CpG Site | Gene | | Mean Difference Methylation | *p*-value | FDR Adjusted *p*-value | Smoke related (Y/N) |
| --- | --- | --- | --- | --- | --- | --- |
| chr6:108882977  chr6:108882982  chr6:108882964  chr6:108882898  chr6:108880383  chr6:108882922  chr6:108882872  chr6:108882916  chr6:108882900  chr6:108882933  chr6:108882914  chr6:108882877  chr6:108883037  chr6:108883006  chr6:108880360  chr6:108882844  chr6:108882941  chr6:108883024  chr6:108882864  chr6:108882852  chr6:108882831  chr6:108882867  chr6:108882825  chr6:108882869  chr6:108879466  chr6:108883485  chr6:108883500  chr6:108882816  chr6:108882819  chr6:108883354  chr6:108883330  chr6:108883503  chr6:108883441  chr6:108883381  chr6:108883281  chr6:108883340  chr6:108883284  chr6:108883399  chr17:108883381  chr6:108883527  chr6:108883287  chr20:32273763  chr6:108883403  chr20:32274387  chr6:108883298  chr3:112281685  chr3:112281810  chr19:41859656  chr20:32273753  chr19:41859677  chr16:55514378  chr19:41858842  chr16:55514466  chr19:41859733  chr20:32273761  chr16:55512868  chr20:32273859  chr16:55514383  chr3:112281831  chr16:55514470  chr16:55514371  chr3:112281636  chr3:112281632  chr6:108879481  chr20:32274289  chr20:32274250  chr20:32274224  chr3:112281821  chr19:41859339  chr3:112281763  chr6:108879805  chr19:41859366  chr3:112280608  chr20:32274325  chr17:7590743  chr19:41859388  chr1:32757717  chr3:112280408  chr19:41858959  chr3:112281767  chr6:108879807  chr19:41859599  chr20:32274110  chr20:32274222  chr6:108879098  chr3:112280580  chr20:32274093  chr3:112280544  chr6:108879674  chr4:75310913  chr3:112280522  chr6:108880486  chr20:32274205  chr4:75310999  chr4:75310908  chr6:108879946  chr20:32274107  chr4:75310841  chr20:32274104  chr1:163292017  chr1:32757818  chr6:108879862  chr19:41858802  chr6:108879506  chr6:108879778  chr17:7591645  chr20:32274113  chr17:7591780  chr20:32274088  chr4:75311092  chr4:75310961  chr3:112281866  chr6:108879516  chr1:163291916  chr4:75480893  chr1:32757731  chr17:7591680  chr4:75311038  chr6:108881587  chr6:108879876  chr6:108879088  chr19:41858740  chr1:32757921  chr1:32757936  chr3:112281638  chr20:32274316  chr1:32757775  chr6:108880604  chr6:108879689  chr1:163291819  chr1:163291557  chr3:112280747  chr4:75310841  chr1:163291918  chr4:75310843  chr20:32274142  chr1:32757936  chr6:108880511  chr6:108879512  chr4:75311104  chr4:75310841  chr20:32274150  chr3:112280738  chr17:7591509  chr19:41858036  chr19:41858823  chr19:41858744  chr19:41858034  chr6:108881578  chr19:41859482  chr6:108879116  chr3:112280603  chr4:75310782  chr6:108879120  chr20:32274079  chr6:108879506  chr4:75311117  chr1:32757756  chr17:7591592  chr3:112280649  chr6:108879083  chr6:108879086  chr19:41858691  chr1:163291565  chr4:75310716  chr19:41858666  chr19:41858622  chr17:7591700  chr6:108879100  chr19:41859500  chr4:75310782  chr17:7591694  chr3:112280605  chr1:32757921  chr6:108879072  chr17:7591553  chr6:108879128  chr4:75310846  chr17:7591753  chr1:32757747  chr4:75310972  chr19:41858683  chr19:41858620  chr6:108879095  chr3:112280608  chr6:108879114  chr17:7591512  chr6:108880317  chr4:75310688  chr19:41857914  chr17:7591731  chr6:108879108  chr1:163291967  chr6:108880536  chr6:108880488  chr19:41858066  chr3:112281626  chr4:75310716  chr19:41858638  chr17:7591768  chr17:7591520  chr4:75310716  chr6:108880412  chr17:7591816  chr3:112280736  chr3:112280786  chr17:7591578  chr17:7591546  chr3:112280636  chr17:7591571  chr19:41857883  chr6:108879081  chr4:75310857  chr3:112280634  chr4:75310702  chr4:75310731  chr1:163291734  chr4:75310729  chr4:75310762 | | FOXO3  FOXO3  FOXO3  FOXO3  FOXO3  FOXO3  FOXO3  FOXO3  FOXO3  FOXO3  FOXO3  FOXO3  FOXO3  FOXO3  FOXO3  FOXO3  FOXO3  FOXO3  FOXO3  FOXO3  FOXO3  FOXO3  FOXO3  FOXO3  FOXO3  FOXO3  FOXO3  FOXO3  FOXO3  FOXO3  FOXO3  FOXO3  FOXO3  FOXO3  FOXO3  FOXO3  FOXO3  FOXO3  FOXO3  FOXO3  FOXO3  E2F1  FOXO3  E2F1  FOXO3  ATG3  ATG3  TGFB1  E2F1  TGFB1  MMP2  TGFB1  MMP2  TGFB1  E2F1  MMP2  E2F1  MMP2  ATG3  MMP2  MMP2  ATG3  ATG3  FOXO3  E2F1  E2F1  E2F1  ATG3  TGFB1  ATG3  FOXO3  TGFB1  ATG3  E2F1  TP53  TGFB1  HDAC1  ATG3  TGFB1  ATG3  FOXO3  TGFB1  E2F1  E2F1  FOXO3  ATG3  E2F1  ATG3  FOXO3  AREG  ATG3  FOXO3  E2F1  AREG  AREG  FOXO3  E2F1  AREG  E2F1  NUF2  HDAC1  FOXO3  TGFB1  FOXO3  FOXO3  TP53  E2F1  TP53  E2F1  AREG  AREG  ATG3  FOXO3  NUF2  AREG  HDAC1  TP53  AREG  FOXO3  FOXO3  FOXO3  TGFB1  HDAC1  HDAC1  ATG3  E2F1  HDAC1  FOXO3  FOXO3  NUF2  NUF2  ATG3  AREG  NUF2  AREG  E2F1  HDAC1  FOXO3  FOXO3  AREG  AREG  E2F1  ATG3  TP53  TGFB1  TGFB1  TGFB1  TGFB1  FOXO3  TGFB1  FOXO3  ATG3  AREG  FOXO3  E2F1  FOXO3  AREG  HDAC1  TP53  ATG3  FOXO3  FOXO3  TGFB1  NUF2  AREG  TGFB1  TGFB1  TP53  FOXO3  TGFB1  AREG  TP53  ATG3  HDAC1  FOXO3  TP53  FOXO3  AREG  TP53  HDAC1  AREG  TGFB1  TGFB1  FOXO3  ATG3  FOXO3  TP53  FOXO3  AREG  TGFB1  TP53  FOXO3  NUF2  FOXO3  FOXO3  TGFB1  ATG3  AREG  TGFB1  TP53  TP53  AREG  FOXO3  TP53  ATG3  ATG3  TP53  TP53  ATG3  TP53  TGFB1  FOXO3  AREG  ATG3  AREG  AREG  NUF2  AREG  AREG | -9.09%  -9.04%  -8.92%  -8.85%  -8.47%  -8.38%  -8.05%  -7.91%  -7.89%  -7.87%  -7.71%  -7.61%  -7.60%  -7.47%  -6.97%  -6.91%  -6.91%  -6.58%  -6.54%  -6.45%  -6.41%  -6.18%  -6.12%  -5.94%  5.69%  -5.58%  -5.10%  -4.96%  -4.84%  -4.46%  -4.28%  -4.26%  -4.15%  -4.11%  -4.09%  -3.98%  -3.95%  -3.85%  -3.83%  -3.63%  -3.54%  -3.45%  -3.29%  2.72%  -2.67%  -2.61%  -1.83%  1.82%  -1.73%  -1.67%  -1.66%  -1.55%  -1.50%  1.46%  -1.45%  -1.24%  -1.16%  -1.14%  1.14%  -1.12%  -1.01%  1.00%  0.97%  -0.95%  0.93%  0.93%  0.91%  0.88%  0.86%  0.82%  0.78%  0.76%  0.75%  -0.72%  -0.68%  0.67%  0.67%  0.62%  0.60%  0.57%  0.55%  0.52%  0.52%  0.51%  0.50%  0.49%  0.49%  0.48%  0.47%  -0.46%  0.46%  0.46%  0.46%  0.46%  0.45%  0.45%  0.44%  0.42%  0.42%  -0.42%  -0.41%  -0.40%  0.39%  0.38%  0.38%  0.37%  0.37%  0.37%  0.35%  0.35%  0.34%  0.34%  0.34%  0.33%  0.33%  0.32%  0.32%  0.32%  0.32%  0.32%  0.31%  0.31%  0.31%  0.31%  -0.31%  -0.31%  0.31%  0.31%  0.31%  0.30%  0.30%  0.30%  0.29%  0.29%  0.29%  0.29%  0.29%  0.29%  0.28%  0.28%  0.28%  0.28%  0.28%  0.28%  0.28%  0.28%  0.27%  0.27%  0.27%  0.27%  0.26%  0.26%  0.26%  0.26%  0.26%  0.26%  0.25%  0.25%  0.25%  0.25%  0.25%  0.25%  0.25%  0.24%  0.24%  0.24%  0.24%  0.24%  0.24%  0.24%  0.23%  0.22%  0.22%  0.22%  0.22%  0.22%  0.22%  0.22%  0.21%  0.21%  0.21%  0.21%  0.21%  0.20%  0.20%  0.20%  0.20%  0.20%  0.19%  0.19%  0.19%  0.19%  0.19%  0.19%  0.19%  0.19%  -0.18%  0.18%  0.18%  0.17%  0.17%  0.17%  0.16%  0.16%  0.16%  0.16%  0.15%  0.15%  0.15%  0.15%  0.15%  0.14%  0.14%  0.13%  0.13%  0.13%  0.13%  0.12%  0.12% | 1.99943E-06  1.53683E-06  6.84495E-05  1.44151E-10  0.002208041  0.002275345  0.00223676  0.003850902  0.000332885  0.00019996  9.38636E-09  0.003055578  1.39361E-05  1.1437E-157  8.31942E-17  3.69906E-10  1.01507E-15  1.5527E-218  1.89189E-10  1.20436E-43  7.90951E-18  2.68065E-06  8.02356E-11  5.23709E-10  1.86502E-46  2.80664E-17  7.95656E-07  5.79674E-13  1.39617E-08  9.6177E-190  1.00051E-15  1.51695E-10  9.50088E-20  5.81402E-19  0.009158671  1.3311E-13  0.001873615  4.0002E-36  0.008623048  6.42752E-18  0.000101598  1.20909E-05  1.2379E-173  0.000144277  0.011063133  0.000907602  0.04597538  0.031488316  0.029452446  0.002275345  6.18328E-09  6.80864E-08  1.27604E-10  5.44212E-07  6.13075E-05  6.82988E-06  1.59758E-05  0.047200853  1.42615E-06  1.2115E-103  1.3213E-128  0.038583274  0.009648634  0.003126022  0.016124415  0.000101598  0.000854782  0.000537718  0.006986467  0.001745512  1.78922E-11  0  0  1.60067E-75  9.68966E-75  9.01934E-85  1.34602E-85  2.90519E-85  8.40337E-81  2.5956E-115  2.4877E-95  2.8003E-79  3.71186E-91  4.97106E-84  1.43143E-89  4.04089E-60  7.20233E-60  2.89827E-67  5.46202E-76  1.1022E-69  8.12584E-59  1.33656E-52  3.13937E-33  2.19255E-41  7.65243E-05  5.55869E-08  1.68948E-07  0.00010891  2.26248E-10  6.32123E-15  8.14276E-08  0.001186471  2.07894E-45  3.67346E-51  9.59806E-40  2.45549E-19  8.40278E-17  4.62649E-51  4.34576E-41  1.12901E-50  8.22621E-61  3.40688E-49  6.28897E-26  7.40968E-20  1.30635E-63  1.77033E-47  2.92762E-38  1.24616E-32  1.96911E-29  0.025650418  4.35346E-06  4.7927E-06  2.30885E-06  0.015846264  0.001321094  0.033158901  0.002825769  1.61056E-52  4.5247E-07  0.000292534  0.004215374  0.035675664  0.001855722  1.92353E-22  3.22987E-10  7.31013E-19  0.042167027  5.41722E-05  1.82216E-05  7.05905E-07  2.37082E-24  0.023484023  0.003055578  0.013069381  3.51638E-06  2.10454E-09  2.0556E-12  1.20994E-16  1.60984E-09  4.99525E-29  0.000358488  2.89526E-11  3.37515E-06  3.22345E-05  0.000429086  1.14523E-09  0.00053934  5.62859E-12  0.000206598  2.68065E-06  1.18475E-08  7.86438E-06  5.28888E-06  5.76444E-08  1.67172E-06  0.046155335  0.003477716  0.006842526  0.00030061  3.13224E-07  2.39869E-11  4.8141E-13  2.89862E-17  0.00279129  1.51196E-05  0.006671081  4.65946E-07  4.63688E-05  1.9317E-05  1.73513E-09  1.36266E-09  0.038013297  0.002882837  0.01936072  7.70745E-05  0.013711793  1.76221E-05  0.000378469  5.76045E-09  0.003319105  0.040852349  0.000907655  6.02793E-07  0.00386647  0.015612199  0.000152172  0.042665308  3.53853E-06  0.01697313  0.004412562  0.04466497  0.001873053  0.044168532  0.000499938  0.03544386  0.001237331  0.00453685  2.68574E-08  4.36742E-29  8.85273E-11  1.69537E-06  1.9931E-08  6.80864E-08  3.41807E-10  1.79583E-08  1.02685E-07  2.44364E-13  2.59059E-12  0.002915662 | 1.68855E-05  3.652E-05  1.67697E-05  2.0834E-05  0.023  1.70413E-05  2.1378E-05  2.2875E-05  1.90123E-05  2.1722E-05  4.1438E-05  1.7864E-05  6.0797E-05  2.2898E-05  0.03  0.000142481  0.0001583  0.00021517  0.001  2.1453E-06  0.001  0.00085526  0.00037704  5.9304E-05  0.031  4.4426E-05  0.001  0.002  0.002  0.0037383  0.00128033  0.002  0.001  0.0049213  0.022976  2.6182E-07  0.001  0.001  0.001  0.004  0.003  0.008  0.006  0.014  0.017  0.037  0.037  0.033  0.01  0.01  0.013  0.041  0.023  0.006  0.046  0.012  0.037  0.027  0.01  0.018  0.045  0.025  0.036  0.007  0.039  0.047  0.032  0.043  0.02  0.03  0.003  0.029  0.021  0.013  0.026  0.01  0.026  0.01  0.019  0.024  0.004  0.004  0.001  0.004  0.002  0.008  0.004  0.044  0.011  0.027  0.015  0.01  0.007  0.011  0.008  0.024  0.009  0  0.007  0.011  0.027  0.007  0.014  0.027  0.037  0  0.013  0.002  0.01  0.016  0.007  0.008  0.003  0.005  0.046  0.047  0.005  0.015  0.018  0.015  0.008  0  0.001  0.004  0.009  0.01  0.014  0.045  0.013  0.035  0  0.002  0.001  0.008  0.012  0.002  0.001  0.006  0.01  0.038  0.015  0.026  0.001  0  0.032  0.006  0.014  0.016  0.032  0.014  0.013  0  0.032  0.018  0.044  0.047  0.043  0.028  0  0.004  0.001  0  0.04  0.041  0.011  0.018  0.013  0.033  0.008  0.045  0.005  0.006  0.005  0.026  0.003  0.002  0.042  0.018  0.009  0.042  0.048  0.041  0.003  0.01  0.015  0.013  0.004  0.049  0.018  0.013  0.037  0.007  0.027  0.03  0.024  0.015  0.005  0.028  0.049  0.014  0.047  0.007  0.046  0.042  0.037  0.018  0.004  0.029  0.042  0.018  0.049  0.047  0.04  0.031  0.048  0.018  0.02  0.008  0.008 | Y  N  Y  Y  Y  Y  Y  Y  N  N  N  N  N  Y  N  Y  N  N  Y  Y  N  N  N  N  Y  N  N  N  Y  Y  N  N  Y  Y  N  N  N  Y  Y  N  Y  Y  N  Y  N  N  N  Y  N  N  Y  N  N  N  Y  N  Y  N  Y  N  N  N  N  N  N  N  N  N  Y  Y  Y  Y  Y  Y  Y  Y  Y  N  Y  N  N  N  Y  Y  N  N  N  Y  N  N  Y  N  N  Y  N  N  N  N  Y  Y  N  N  N  N  N  N  Y  N  N  N  Y  N  Y  N  Y  Y  Y  Y  Y  Y  Y  N  N  Y  Y  Y  N  N  N  Y  N  Y  N  N  Y  N  N  N  N  N  N  N  N  N  N  N  N  N  N  Y  N  N  N  N  N  N  N  Y  Y  N  N  N  Y  Y  N  Y  Y  N  N  Y  Y  Y  N  N  Y  N  Y  N  Y  N  N  N  N  N  N  N  N  N  N  N  N  N  N  N  N  N  N  N  N  N  N  N  N  N  N  N  N  N  N  N  N  N  N  N  N  N  N  N  N |
|  |  | |  |  |  |  |
